# Efficient single photon source based on μ-fibre-coupled tunable microcavity

**DOI:** 10.1038/srep14309

**Published:** 2015-09-22

**Authors:** Chang-Min Lee, Hee-Jin Lim, Christian Schneider, Sebastian Maier, Sven Höfling, Martin Kamp, Yong-Hee Lee

**Affiliations:** 1 Department of Physics, KAIST, Daejeon 305-701, South Korea; 2 Technische Physik, Physikalisches Institut and Wilhelm Conrad Röntgen-Research Center for Complex Material Systems, Universität Würzburg, Am Hubland, D-97074, Würzburg, Germany; 3 SUPA, School of Physics and Astronomy, University of St. Andrews, St. Andrews, KY 16 9SS, UK

## Abstract

Efficient and fast on-demand single photon sources have been sought after as critical components of quantum information science. We report an efficient and tunable single photon source based on an InAs quantum dot (QD) embedded in a photonic crystal cavity coupled with a highly curved μ-fibre. Exploiting evanescent coupling between the μ-fibre and the cavity, a high collection efficiency of 23% and Purcell-enhanced spontaneous emissions are observed. In our scheme, the spectral position of a resonance can be tuned by as much as 1.5 nm by adjusting the contact position of the μ-fibre, which increases the spectral coupling probability between the QD and the cavity mode. Taking advantage of the high photon count rate and the tunability, the collection efficiencies and the decay rates are systematically investigated as a function of the QD–cavity detuning.

On-demand single photon sources (SPSs) are crucial components for quantum information processing (QIP) such as quantum cryptography^1^, linear optic quantum computing^2^, and quantum memory^3^. For the practical use in QIP, SPSs should have high source efficiency and high speed. A single semiconductor quantum dot (QD) is a promising quantum emitter^4^ because of its integrability into other semiconductor devices to build a scalable quantum circuit. However, it is difficult to extract photons with a high efficiency, since the QD is embedded in a high-refractive-index semiconductor. Besides, the speed of the QD SPS is limited by the spontaneous emission (SE) lifetime of the exciton (order of 1 ns). To realize efficient and fast QD SPSs, there has been much effort to engineer photonic nanostructures such as micropillars^5^,^6^,^7^,^8^, nanowires^9^,^10^,^11^,^12^,^13^, photonic crystal (PhC) waveguides^14^,^15^,^16^, and PhC cavities^17^,^18^,^19^.

PhC cavities provide high quality factors and small mode volumes simultaneously^20^,^21^, which enables pronounced SE rate enhancement via the Purcell effect^22^,^23^,^24^. They also offer high single-mode coupling efficiency (SE factor) owing to a photonic bandgap, which suppresses SE into the other photonic modes near the resonance. However, there are a few obstacles to realizing efficient and fast QD SPS with high-quality PhC cavities. Because of narrow bandwidth of the QD emission and the PhC cavities, it is challenging to spectrally couple the QD and the cavity mode. For high-quality PhC cavities, wavelength tunability is essential to make the coupling feasible. Besides, the small volume of the PhC cavity results inevitably in highly divergent output beams and hinders efficient photon collection. To avoid those difficulties, coupling schemes based on a μ-fibre and the PhC cavity have been demonstrated^25^,^26^,^27^.

In this work, an efficient fibre-coupled SPS is demonstrated using a highly curved μ-fibre and a PhC cavity with an embedded QD. The curved μ-fibre is employed as both an efficient photon-funnelling channel and a cavity-tuning method. Using the μ-fibre, a large photon count rate of 300 kHz is obtained at single photon (SP) detectors. The estimated SP count rate (collection efficiency) at the μ-fibre is 18 MHz (23%). The high total collection efficiency stems from both the high fibre coupling efficiency of this system (41%) and the high SE factor (>0.82) of the PhC cavity. The μ-fibre-coupled microcavity is tunable by changing the fibre contact position, originated from an effective refractive index change^28^. This tuning method is rapid compared to other tuning methods^29^,^30^. Owing to the high SP count rates and the tunability, we were able to study collection efficiencies and radiative lifetimes at different detunings between the QD emission and the cavity mode. Compared to the previous fibre-coupled SPSs with broadband coupling scheme^31^,^32^,^33^,^34^, our fibre-coupled PhC cavity exploits Purcell effect and high single-mode coupling efficiency. Equipped with high efficiency and speed, the curved μ-fibre-coupled PhC cavity with a QD is a suitable platform to be implemented in QIP.

## Results

### μ-fibre-coupled photonic crystal cavity

**Figure 1:**
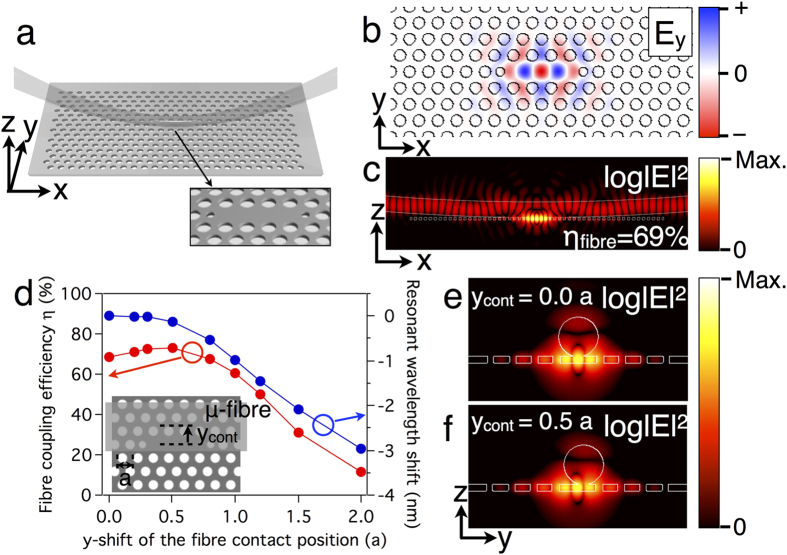
Characteristics of a μ-fibre-coupled photonic crystal (PhC) cavity. (**a**) A schematic of a curved microfibre-coupled PhC L3 cavity. Inset: magnified image of the cavity region. (**b**) Calculated electric field (E_y_) profile of the cavity mode of interest. (**c**) Calculated electric field intensity profile at the y = 0 plane. (**d**) The fibre coupling efficiency η and the resonant wavelength shift as functions of y_cont_. (**e**,**f**) Calculated electric field intensity profiles at the x = 0 plane, when (**e**) y_cont_ = 0.0 *a* and (**f**) y_cont_ = 0.5 *a*.

A curved μ-fibre-coupled PhC linear three-cell (L3) cavity, shown in Fig. 1a, is investigated. Air holes at both sides of the cavity are reduced and shifted to alleviate cavity losses^20^. The contact between the μ-fibre and PhC slab is robust owing to the electrostatic force between them. InAs/GaAs QDs are embedded in the slab as quantum emitters. In this structure, the overall single photon collection efficiency ξ through the curved μ-fibre is defined as a ratio of the collected SP count rate to the repetition rate of pump pulses, and it is expressed as ξ = βη, assuming that the internal quantum efficiency of the QD is unity^8^,^35^. Here, β is the SE factor, and η is the coupling efficiency between the cavity mode and the μ-fibre mode. The SE factor is expressed as 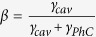, where γ_cav_ and γ_PhC_ are SE rates to the cavity mode and to the other modes, respectively. γ_cav_ is expressed as


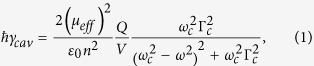


and its enhancement via the Purcell effect^36^,^37^ is quantified as


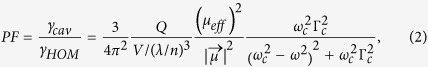


where Q and V are the quality factor and the mode volume of the cavity mode, respectively; ε_0_n^2^ is the dielectric permittivity for the cavity photon; ω and ω_c_ are the frequencies of an emitter and the cavity, respectively; and Γ_*c*_ is the linewidth of the cavity mode. μ_eff_ is an effective dipole moment coupled with a cavity mode, which is defined by


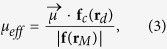


where **f**_*c*_(**r**_*d*_) and **f**_*c*_(**r**_*M*_) are cavity electric fields at the QD position and the position where the energy density is maximum, respectively; 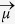 is a transition dipole moment of the QD. A Rabi frequency^38^ g_c_ is proportional to the effective dipole moment as


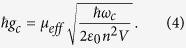


The ratio 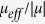 and *g*_*c*_ quantify how effectively and how strongly the QD is coupled to the cavity, respectively. A coupling of the QD with the cavity mode and a sufficient Q/V for γ_cav_ to overwhelm γ_PhC_ make the PhC cavity avail of the high β factor. Accompanying this feature, the high η of the curved μ-fibre-coupled L3 cavity structure assists in realizing efficient SPSs.

Numerical simulations employing finite-difference time-domain (FDTD) methods are performed to understand the μ-fibre-coupled PhC cavity. Mode profiles are similar to that of a typical unperturbed L3 cavity (Fig. 1b). The fibre coupling efficiency η is obtained by the ratio between the fibre-coupled Poynting flux and the total generated Poynting flux. PhC design parameters are chosen to maximize η. When the μ-fibre is in contact with the optimized cavity at the centre (y_cont_ = 0.0 *a*) (*a*: lattice constant), the fibre coupling efficiency η is 69% (Fig. 1c).

To check the stability, the fibre coupling efficiency is calculated for different fibre contact positions (*y*_*cont*_) along the vertical direction (Fig. 1d). When the contact position is slightly off from the centre (*y*_*cont*_ = 0.5 *a*), η increases to 73%. This is understandable if we see the mode profile in the YZ plane (Fig. 1e,f). The electric field intensity lobes next to the central lobe have longer evanescent tails in the z-direction. Therefore, when we move the μ-fibre, an overlap between the cavity mode and the μ-fibre can be slightly increased. If we plot the quality factor of each loss channel as a function of y_*cont*_, Q_fibre_ reaches a minimum around y_*cont*_ = 0.5 *a*, which supports the analysis [see Supplementary Information]. The fibre coupling efficiency η is maintained above 50% within *y*_*cont*_ = 1.2 *a*. In the same range of *y*_*cont*_ = 1.2 *a*, the resonant wavelength of the cavity mode blueshifts by 1.5 nm due to the decrease of effective refractive index of the cavity mode. In other words, we can tune the cavity resonance by moving the contact position of the μ-fibre. Note that the total cavity loss remains nearly unchanged throughout the tuning process, which will be discussed more with the experimental data.

### Efficient μ-fibre-coupled single photon source

**Figure 2:**
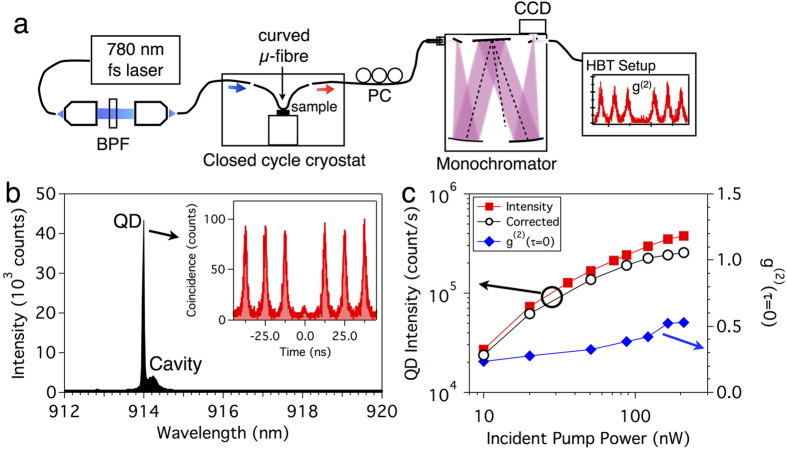
Efficient single photon collection. (**a**) A photoluminescence (PL) measurement setup. BPF: band-pass filter, PC: polarization controller. (**b**) A PL spectrum. The cavity mode is detuned by 0.2 nm from the QD emission wavelength. Linewidth of the QD emission line is less than spectral resolution of our setup (0.07 nm). Inset: measured coincidence of the QD peak at 10 nW pump power, with a 0.1 nm spectral window. (**c**) Detected and corrected single photon count rates and g^(2)^(0) as functions of the incident pump power.

Single photons are generated and collected in a fibre-coupled μ-photoluminescence (μ-PL) setup at 20 K (Fig. 2a and see Methods). A 780-nm femtosecond laser with an 80-MHz repetition rate is used as a pump source. The pump laser is focused to one end of the curved μ-fibre and absorbed by the QD, which has been placed in a closed cycle cryostat. Generated SPs are collected by the same μ-fibre and directed to both arms of the curved μ-fibre. Collected SPs are spectrally filtered by a 0.5-m-long monochromator and detected by a charge-coupled device or a Hanbury Brown and Twiss (HBT) measurement setup with two single photon detectors. Note that the generated SPs are maintained in the fibre except during the spectral filtering. To estimate the μ-fibre coupling efficiency ξ, we calibrated the transmission of each component and detection efficiencies of single photon detectors. The total detection efficiency, from the μ-fibre to the single photon detectors, is 2.5% [see Supplementary Information].

A single QD spectrally close to the cavity resonance (QD1) is selected to study the fibre collection efficiency ξ and the SE lifetime. A normalized detuning δ_norm_ is defined as δ_norm_ = (λ_cav_ − λ_QD_)/Δλ_cav_, where Δλ_cav_ is the cavity linewidth. A typical PL spectrum is shown in Fig. 2b; the normalized detuning δ_norm_ = 0.88. In this case, typical coincidence counts from the HBT setup (Fig. 2b, inset) show strong antibunching behaviours. The value of g^(2)^(0) is the ratio of the area of the central peak to the area of the other peaks.

Both SP count rates and second-order autocorrelations are measured as a function of pump power to estimate the total collection efficiency ξ (Fig. 2c). When a QD is coupled to a nanocavity, the emitted SP stream is considered as a mixed state of a pure SP state and coherent states from the background^5^. Therefore, we corrected the detected SP count rates by multiplying 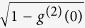 to compensate for the background contributions. When g^(2)^(0) reaches 0.42, the detected SP count rate is 300 kHz and the corrected SP count rate is 230 kHz. Assuming that the same number of SPs are emitted into the other arm of the fibre and taking the total detection efficiency into account, the estimated total SP count rate (collection efficiency ξ) is 18 MHz (23%). Besides, we estimated a fibre coupling efficiency η of 41% from transmission measurement with a broadband light source [see Supplementary Information]. We attribute the difference between two efficiencies ξ and η to both the β factor and internal quantum efficiency. We emphasize that the generated single photons are already in an optical fibre and ready for further processing.

**Figure 3:**
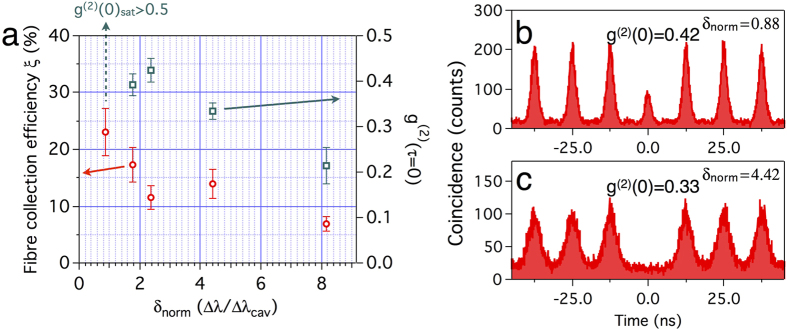
Collection efficiencies and coincidences at different detuning. (**a**) Estimated total fibre collection efficiency and g^(2)^(0) at different δ_norm_. Error bars include fluctuation of the detectors and errors in transmission measurements of the setup. (**b**,**c**) Coincidence counts at saturation pump power when (**b**) δ_norm_ = 0.88, (**c**) δ_norm_ = 4.42. Integration times for (**b**,**c**) are 30 s and 120 s, respectively.

Similarly, the collection efficiencies ξ and g^(2)^(0) are measured as a function of detuning (Fig. 3a). The resonant wavelength of the cavity mode is tuned by digital etching^29^ from δ_norm_ = 8.15 to δ_norm_ = 0.88, and by gas deposition techniques^30^ to longer wavelengths. The fibre contact position remains near the centre of the cavity (*y*_*cont*_ = 0.5 *a*) throughout the experiment. As the cavity mode moves further from the QD emission wavelength, the estimated collection efficiency gently decreases. This trend comes from the detuning dependence of the β factor. However, even when the cavity mode is far away from the QD emission (δ_norm_ = 8.15), the measured fibre collection efficiency ξ is over 5%, which is comparable to other fibre-coupled SPSs^31^,^32^,^33^.

For high pump powers at which the QD is saturated, second-order autocorrelations are shown in Fig. 3b,c. When δ_norm_ = 0.88, g^(2)^(0) reaches 0.42 and keeps increasing with the incident pump power. Note that coincidence counts from dark count of the detectors or background PL which is not related to the cavity mode are negligibly small. At this stage, both the sharp peak and overall background contribute to the finite g^(2)^(0) value. The peak at τ = 0 has several origins, one of them being recapture processes^39^,^40^. As the radiative lifetime is shortened by the Purcell effect, the recapture becomes more probable. The other origin is the cavity-enhanced SE from continuum states of the QD that have the same wavelength with the QD exciton. Continuum states from Coulomb interaction between multiple electrons and holes feed the cavity mode off-resonantly^41^,^42^,^43^,^44^, then it contaminates the purity of the single photons. When the cavity mode moves further from the QD emission, the peak at τ = 0 vanishes because of suppressed recapture processes and the spectral filtering of cavity-enhanced continuum states (Fig. 3c). However, the overall background still remains and increases the multiphoton probability, which is asynchronous with the pump pulse^35^,^39^,^44^,^45^. A plausible scenario is random recaptures of charge carriers stored in charge traps near the QD^45^. In our case, if the QD is located near the surface of the air holes, the surface can act as the charge traps. The smaller g^(2)^(0) could be obtained by resonant pumping^39^, or by temporal filtering at the expense of the SP count rate^35^.

### Purcell-enhanced spontaneous emission rate and its detuning dependence

**Figure 4:**
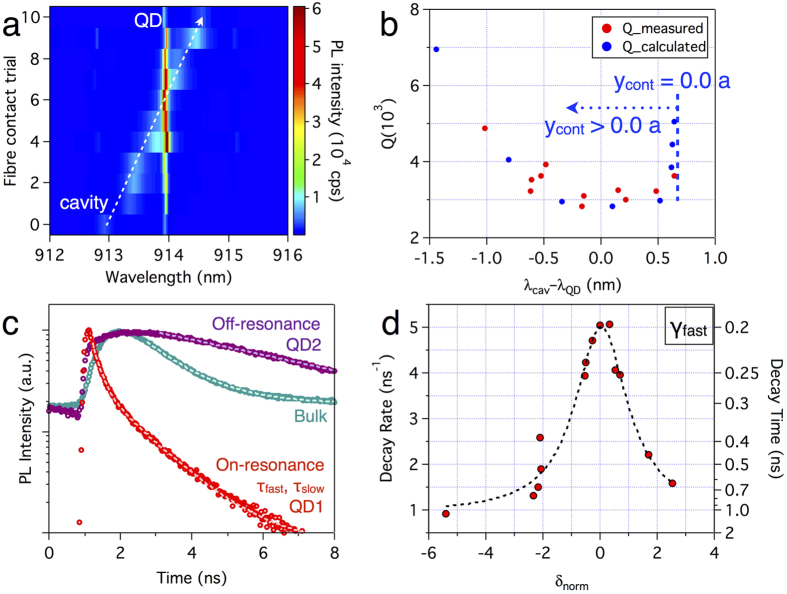
Purcell-enhanced SE rate and its detuning dependence. (**a**) Photoluminescence (PL) spectra at the different fibre contact positions. Resonant wavelength is tuned by as much as ~1.5 nm. (**b**) Measured quality factor and calculated quality factor as functions of detuning. (**c**) Time-resolved PL for different resonance conditions. Dotted lines are fit with a biexponential (red) or exponential (green and purple) function. (**d**) Decay rates of the fast component at different detuning. Black dotted line is a Lorentzian fit.

From the numerical simulations (Fig. 1d), we found that the resonant wavelength of a cavity mode is tunable by moving the contact position of the μ-fibre. The spectral tunability is confirmed experimentally by measuring PL spectra as we vary the fibre contact position (Fig. 4a). For each PL measurement, the PhC cavity is re-positioned using piezoelectric nanopositioners. Through this method, the cavity resonance is tuned by as much as 1.5 nm (5–8 times Δλ_cav_). However, this tuning process disturbs the cavity and changes the quality factor. The quality factor Q = λ_cav_/Δλ_cav_ of Fig. 4b is plotted using Δλ_cav_ measured in Fig. 4a. Throughout the tuning range, the quality factors remain between 3000 and 5000, which makes it possible to study detuning-dependent QD lifetimes. As the μ-fibre contact position moves away from the centre of the cavity, the quality factor decreases slightly due to increased coupling losses into the μ-fibre. When the μ-fibre moves even further, the quality factor increases again because it becomes the unperturbed L3 cavity. Calculated quality factors from the FDTD simulation support the measured trend.

Lifetimes of QDs under different resonance conditions are measured through time-resolved PL (Fig. 4c). The lifetime of the QD ensemble in bulk is determined to be 1.23 ns. The decay of the on-resonant QD1 shows biexponential behaviours with τ_fast_ = 200 ps, τ_slow_ = 1.09 ns. The fast component is associated with radiative decay. It shows clear SE rate enhancement by the Purcell effect. The slow components are ascribed to several origins, such as the background emission coupled into the spectral window, or the spin-flip transition from dark to bright exciton. For the detuning-dependent experiment of Fig. 4d, we concentrate on the fast radiative component. The lifetime of another QD (QD2) that is spectrally far detuned from the cavity mode (δ_norm_ = 22.9) in a different cavity is 6.4 ns, which is much larger than the bulk lifetime.

We study SE rates at different detunings by employing the fibre position tuning method (Fig. 4d). The decay rate γ_fast_ is maximal at the resonance (δ_norm_ = 0). However, the spectral dependence of the decay rate obtained from the Lorentzian fit is 2.2 times broader than the cavity linewidth. We attribute this to phonon-assisted transitions between excitons and the cavity mode. Since our measurement is performed at 20 K, both the phonon emission and absorption cause the transition and make the spectral dependence broad and symmetric^46^. Nevertheless, the high SP count rate and the rapid tuning method enable us to study the detuning dependence at constant temperature.

From the measurement, we can estimate the β factor and Purcell enhancement. γ_cav_ is estimated to be 4.1 ns^−1^ from the SE rate when δ_norm_ ~ 0. Meanwhile, at the maximum detuning we obtained (δ_norm_ = −5.4), an upper bound of the γ_PhC_ can be determined to be 0.91 ns^−1^. Therefore, the β factor is >0.82 for QD1 on resonance. Note that from the detuning dependence with rapid tuning method, we could measure SE rates of the same QD (QD1) at different detuning. The Purcell enhancement is lower than expected (Eq. (2)) because it is not easy to find a right QD coupled well with the cavity field. For our case of QD1, μ_eff_/|μ| is estimated to be 0.11, noting that g_c_ = 10 GHz (μ_eff_ = 7 Debye) inferred by Eq. (4) is not as high as precedent studies^41^,^47^ had achieved.

## Discussion

In summary, a μ-fibre-coupled SPS based on a PhC cavity with an embedded QD is demonstrated. High fibre coupling efficiency and the tunability are predicted by FDTD simulation. From the measured SP count rate of 300 kHz, total fibre collection efficiency ξ is estimated to be 23%; it remains over 5% until the cavity mode is detuned by 8 times the cavity linewidth. Fibre collection efficiency η is estimated to be 41% from the transmission measurement. On-resonant QD lifetime is measured to be 200 ps, which shows strong Purcell enhancement compared to the QD lifetime in bulk GaAs. By changing the μ-fibre contact position, the detuning dependence of the decay rate is also investigated.

Even though we obtained 23% SP collection efficiency directly into the μ-fibre, we did not reach the maximum efficiency of this system, which is the measured coupling efficiency η = 41%. The crucial hindrance was a degraded SE factor. Since the QD is not located at the energy maximum of the cavity mode, both the SE factor and Purcell factor are degraded compared to the calculation. Therefore, if QD positioning and aligned lithography techniques were used to locate the QD at the right position^41^,^48^, a near-unity β and a higher ξ approaching 70% could be expected.

Still, the curved μ-fibre-coupled PhC cavity system has strong points. It shows an efficient collection of single photons with a Purcell-enhanced SE rate. In addition, the tunability of this system (~1.5 nm) increases the spectral matching probability of the QD and the cavity by several times. Considering its principle, the tunability is also expected for the other kinds of PhC cavities, which are coupled to a μ-fibre. We finally emphasize that the direct fibre collection of single photons is advantageous for both connecting to other optical components and transmitting over long distances. The total collection efficiency of 23% directly into the single mode fibre compares well with other efficient SPSs^8^,^10^,^49^.

## Methods

### Sample fabrication

Our QD wafer is grown by molecular beam epitaxy (MBE). InAs QDs with a density of about 1 × 10^9^ cm^−2^ are embedded in a 125-nm-thick GaAs slab grown on top of an Al_0.8_Ga_0.2_As sacrificial layer. PhC cavities are fabricated based on the optimized design parameters obtained from numerical simulations. PhC patterns are defined by e-beam lithography followed by Cl_2_-assisted argon ion beam etching. Selective wet etching with hydrogen fluoride solution is performed to remove the sacrificial layer. Lattice constant *a*, hole radius *r*/*a*, side hole radius *rs*/*a*, side hole shift *s*, and slab thickness *t* are 256 nm, 0.32 *a*, 0.25 *a*, 0.2 *a*, and 125 nm, respectively. To fabricate the curved μ-fibre, a conventional optical single-mode fibre is tapered with flame-brushing techniques^50^ down to a diameter of 0.8 μm, then bent such that the radius of curvature is ~100 μm. The Supplementary Information includes the figures for the fabricated PhC cavity and the curved μ-fibre.

### Optical measurements

The PhC sample is loaded inside a closed cycle cryostat. It is controlled by piezoelectric nanopositioners with resolution better than 10 nm. The curved μ-fibre is also loaded inside the cryostat and controlled with DC motors outside. For all optical measurements, a 780-nm femtosecond laser with an 80-MHz repetition rate is used for a pump source, which pumps above the bandgap of GaAs. The pump laser and the generated PL are collected by the same μ-fibre, except for the case of the QD lifetime measurement in bulk. In the bulk measurement, the pump laser is incident obliquely and the generated signals are collected through a 50× microscope objective with a long working distance (N.A. = 0.42). The collected PL is filtered via a spectrometer, whose spectral resolution is ~0.07 nm. To measure SP count rate and coincidence, we use single photon avalanche diodes (SPADs) with relatively high efficiency (~20%) and slow response time (τ ~ 400 ps). To measure the QD lifetime, we use a less efficient, faster (τ ~ 40 ps) SPAD. For the bulk lifetime measurement, the efficient and slow SPAD is used due to the low collection efficiency.

## Additional Information

**How to cite this article**: Lee, C.-M. *et al.* Efficient single photon source based on µ-fibre-coupled tunable microcavity. *Sci. Rep.* **5**, 14309; doi: 10.1038/srep14309 (2015).

## Supplementary Material

Supplementary Information
