# Supplementary material for: Efficient single photon source based on μ-fibre-coupled tunable microcavity
Source: Sci Rep. 2015 Sep 22;5:14309. doi: 10.1038/srep14309 (PMC4585754; doi:10.1038/srep14309)
Supplement: Supplementary Information [file srep14309-s1.pdf]

Supplementary Information for

Efficient single photon source based on μ-fibre-coupled tunable microcavity

Chang-Min Lee,[1,4] Hee-Jin Lim,[1,4] Christian Schneider,[2] Sebastian Maier,[2] Sven Höfling,[2,3] Martin Kamp,[2] and Yong-Hee Lee[1,*]

[1]*Department of Physics, KAIST, Daejeon 305-701, South Korea*

[2]*Technische Physik, Physikalisches Institut and Wilhelm Conrad Röntgen-Research Center for Complex Material Systems, Universität Würzburg, Am Hubland, D-97074, Würzburg, Germany*

[3]*SUPA, School of Physics and Astronomy, University of St. Andrews, St. Andrews, KY 16 9SS, UK*

[4]*These authors contributed equally.*

[*]*E-mail : yhlee@kaist.ac.kr*

1. Quality factors of each loss channel

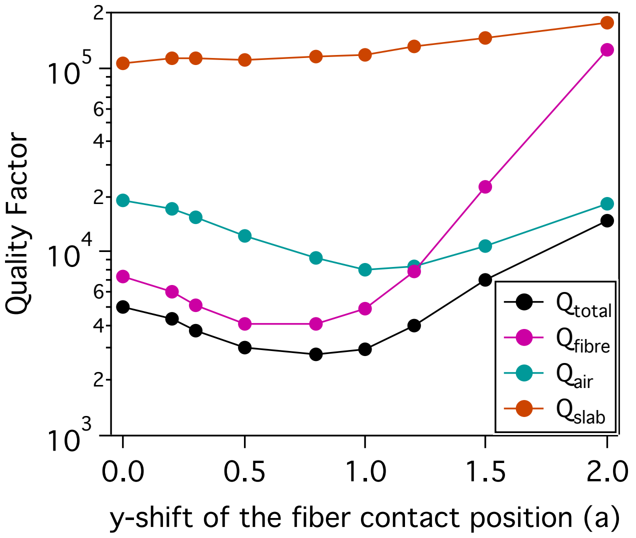

Figure S1. Calculated quality factors corresponding to each loss channel of the fibre-coupled photonic crystal cavity as a function of fibre contact position.

We performed numerical simulations using finite-difference time-domain(FDTD) method to investigate losses of the μ-fibre-coupled photonic crystal cavity. There are three loss channels, which are fibre, slab, and air. Total quality factor Q is obtained by Fourier transformation of the steady state cavity field. Q of each loss channels are obtained by $Q_{channel} = Q_{total} \frac{P_{total}}{P_{channel}}$, where P is the Poynting flux. Thus, the fibre coupling efficiency η is determined to be Q_{total} / Q_{fiber}. As we change the fibre contact position(y_{cont}), the loss to the fibre and air are simultaneously increased. The fibre loss has its maximum around $y_{cont} = 0.5\ a$, which supports the analysis of Fig. 1d-f. The air loss has its maximum around $y_{cont} = 1.0\ a$ and dominates the other losses as the y_{cont} increases more.

2. Figures of the fabricated samples

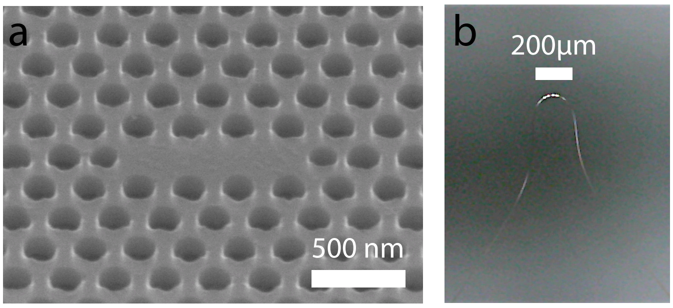

Figure S2. (a) Scanning electron micrograph of a fabricated photonic crystal L3 cavity. (b) Optical image of a curved μ-fibre. Radius of curvature is ~ 100 μm.

3. Detection efficiency of the measurement setup

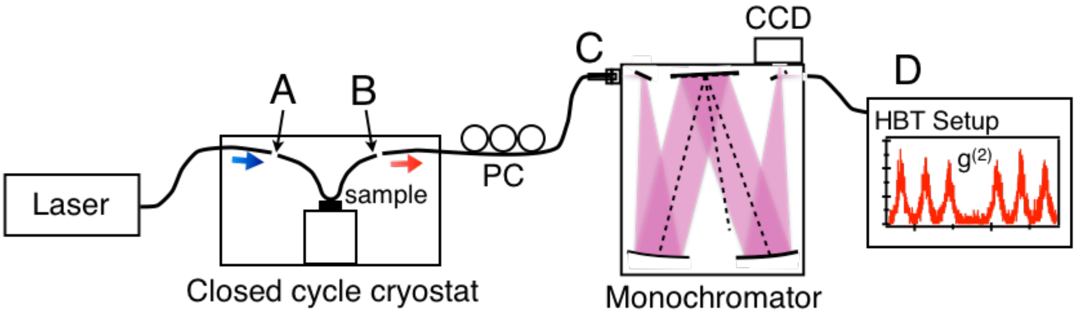

Figure S3. The measurement setup indicating the points at which transmitted powers are detected.

We measured the transmission of each component and the detection efficiency of the single photon detectors to calibrate the measurement setup. We used a continuous-wave laser with narrow linewidth whose wavelength is near the QD emission.

(1) Transmission of the μ-fibre(P_B/P_A) : 40%. Therefore, the transmission between the taper waist and the output arm(B) is estimated as $\sqrt{0.4} = 63\%$.

(2) Transmission of the fibre connector(P_C/P_B) : 90%.

(3) Transmission of the monochromator(P_D/P_C) : 20%.

(4) Detection efficiency of the single photon detectors : 22%.

Therefore, total detection efficiency from the taper waist(in contact with the PhC cavity) to the single photon detectors is $0.63 \times 0.90 \times 0.20 \times 0.22 = 2.5\%$.

4. Transmission measurement

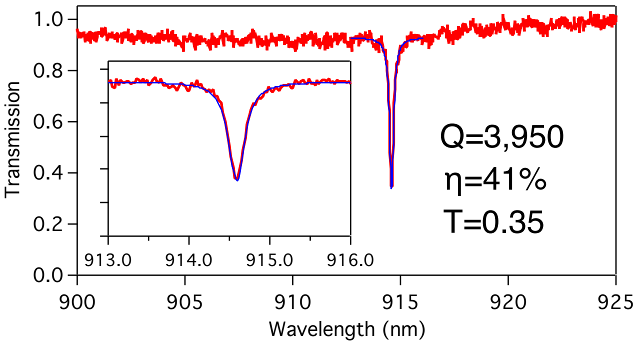

Figure S4. A transmission spectrum of the fibre-coupled photonic crystal cavity. Broadband light source is incident to one arm of the fibre, and the transmitted light is measured from another arm of the fibre. A cavity mode is confirmed at 914.5 nm. The transmission dip is fitted with a coupled mode theory to obtain Q-factor (blue line). Inset : same transmission spectrum with magnified x-scale between 913 nm to 916 nm.

We measure a transmission spectrum of the fibre-coupled photonic crystal cavity. A broadband light source (halogen lamp) is used instead of the laser in Fig. S3. Transmitted light is measured with the spectrometer and CCD. The measured transmission spectrum is shown in Fig. S4. By fitting the transmission dip with coupled mode theory, quality factor of 3,950 and fiber coupling efficiency η of 41% are obtained.
